# Interaction Between MDSC and NK Cells in Solid and Hematological Malignancies: Impact on HSCT

**DOI:** 10.3389/fimmu.2021.638841

**Published:** 2021-02-12

**Authors:** Nicola Tumino, Anna Laura Di Pace, Francesca Besi, Linda Quatrini, Paola Vacca, Lorenzo Moretta

**Affiliations:** Immunology Research Area, IRCCS Bambino Gesù Children's Hospital, Rome, Italy

**Keywords:** natural killer cells, myeloid-derived suppressor cells, hematopoietic stem cell transplantation, tumor microenvironment, hematological malignancies

## Abstract

Myeloid derived suppressor cells (MDSC) are heterogeneous populations that through the release of soluble factors and/or by cell-to-cell interactions suppress both innate and adaptive immune effector cells. In pathological conditions, characterized by the presence of inflammation, a partial block in the differentiation potential of myeloid precursors causes an accumulation of these immunosuppressive cell subsets both in peripheral blood and in tissues. On the contrary, NK cells represent a major player of innate immunity able to counteract tumor growth. The anti-tumor activity of NK cells is primarily related to their cytolytic potential and to the secretion of soluble factors or cytokines that may act on tumors either directly or indirectly upon the recruitment of other cell types. NK cells have been shown to play a fundamental role in haploidentical hemopoietic stem cell transplantation (HSCT), for the therapy of high-risk leukemias. A deeper analysis of MDSC functional effects demonstrated that these cells are capable, through several mechanisms, to reduce the potent GvL activity exerted by NK cells. It is conceivable that, in this transplantation setting, the MDSC-removal or -inactivation may represent a promising strategy to restore the anti-leukemia effect mediated by NK cells. Thus, a better knowledge of the cellular interactions occurring in the tumor microenvironment could promote the development of novel therapeutic strategies for the treatment of solid and hematological malignances.

## Introduction

Tumor microenvironment (TME) consists of an assortment of tumor and non-tumor cells (including mesenchymal stromal cells, endothelial cells, regulatory T-cells, and myeloid-derived suppressor cells), as well as soluble components. Tumor associated (TA)-cells may favor neoplastic transformation, tumor growth, and metastasis thus contributing to tumor escape from host immunity. In addition to TA-cells, TME also contains immune cells including innate and adaptive lymphocytes. A growing body of evidences has revealed the existence of a close relationship between tumor and immune components. Many of the interactions between TME and tumor infiltrating (TI) immune cells are already well-known (1). Consistent positive prognostic correlations have been reported for T-cells, especially cytotoxic T-cells, in different tumor types (2). In particular, TI-lymphocyte density can influence prognosis within each tumor, lymph node, and metastasis (TNM) stage, complementing or even outperforming pathological criteria alone, as shown in colorectal and lung cancers (3–5). In order to develop strategies to overcome immunosuppression and tumor escape it is important to further unravel the cellular interactions occurring in TME. This contribution is focalized on the polymorphonuclear (PMN)-MDSC, an important, strongly immunosuppressive myeloid component, which may greatly impair the anti-tumor defenses in particular those mediated by NK cells.

### MDSC in Physiological and Pathological Condition

One important cellular subset present in TME is represented by MDSC. These cells were first identified in 1970 as a heterogeneous group of immune cells with immature features derived from a common myeloid progenitor (CMP) (6). They possess high immunosuppressive and pro-tumorigenic capabilities and actively cooperate with other myeloid regulatory cells as tumor-associated neutrophils (TANs), tumor-associated macrophages (TAMs), and regulatory dendritic cells to favor cancer development (7). MDSC are usually classified in two or three classes in mice and humans, respectively. In mice, MDSC are classified into monocytic (M-MDSC) (CD11b^+^Ly6C^hi^Ly6G^−^) and PMN-MDSC (CD11b^+^Ly6C^lo^Ly6G^+^ cells) subsets. In humans, the granulocytic and the monocytic subsets are classified as Lin^−^(CD3, CD19, CD56), CD11b^+^CD33^+^CD15^+^CD66b^+^CD14^−^HLA-DR^low/−^ and Lin^−^CD11b^+^CD33^+^CD15^−^CD14^+^HLA-DR^low/−^cells, respectively. A new subset of immature and early-stage MDSC (e-MDSC) has been found in humans and classified as Lin^−^ HLA-DR^low/−^ CD11b^+^ CD14^−^ CD15^−^ CD33^+^. Recently, the lectin-type oxidized LDL receptor-1 (LOX-1) molecule has been suggested as novel marker to discriminate MDSC from neutrophils (8–10).

In physiological condition, the CMP differentiate into neutrophils or monocytes. In pathological conditions such as cancer or infection the inflammatory milieu (e.g., GM-CSF, TNF-α, VEGF, and PGE2) induces the CMP differentiation into M-MDSC, and subsequently TAM, or PMN-MDSC that can differentiate into TAN. In addition, chronic inflammatory conditions induce release of soluble mediators that are responsible for MDSC accumulation due to their reduced susceptibility to Fas-mediated apoptosis (11). In this setting, MDSC can help to control excessive inflammation, by reducing both innate and adaptive immune responses (12, 13), while their reduction normally occurs following inflammation resolution. Conversely, if MDSC numbers do not decrease, they can be associated to disease progression. Indeed, a continuous inhibitory effect of immune response can interfere with tissue homeostasis, energy metabolism, and dead cell removal.

In cancer, MDSC activity is mainly regulated by three key events: myelopoiesis impairment, MDSC migration to tumor site and subsequent activation. Thus, deregulated myelopoiesis leads to accumulation of immature MDSC in bone marrow that are subsequently recruited to primary and metastatic tumor sites by tumor-released chemokines. Due to their potent immunosuppressive and pro-tumorigenic potential, high levels of MDSC, especially PMN-MDSC, have been observed in high grade cancers and are correlated with poor prognosis, treatment resistance and reduced overall survival in solid cancers (14–18). Breast, ovarian and gastric cancer cells secrete CCL2 that recruits MDSC and sustains tumor growth (19). In addition, CCR2^+^MDSC can support tumor growth in colon-rectal-cancer (CRC)-bearing mice (20). CXCL1 is another cytokine, highly expressed in CRC that exerts chemoattractant activity on CXCR2^+^MDSC. Moreover, MDSC also express CCR5 that has been suggested to be involved in MDSC migration to tumor site (21, 22). Regarding MDSC recruitment, it has been demonstrated that the hematopoietic 5-lipoxygenase (5LO), a metabolite of the arachidonic acid implied in colon carcinogenesis, is involved into MDSC migration (23). In addition, MDSC can regulate and induce their recruitment by a positive feedback. Indeed, Reactive Nitrogen Species produced by MDSC lead to chemokine nitration that in turn recruit MDSC (24).

Following recruitment, MDSC are activated through many mechanisms. In particular, PGE2 can induce STAT3 phosphorylation that mediates both MDSC activation (25) and inhibition of their physiological differentiation toward neutrophils or monocytes (26, 27). Histamine can modulate the expression of Arginase-1 (ARG-1) and Nitric oxide synthase (iNOS) in M-MDSCs and PMN-MDSC, respectively (28, 29). Hypoxia can induce the Hypoxia-inducible Factor 1 alpha (HIF-1α) that in turn increases ARG-1 and iNOS activation in MDSC (30).

While many studies on the involvement of MDSC in hematological disorders have been performed, their actual role is still debated. High numbers of PMN-MDSC were reported in chronic myeloid leukemia (CML), possibly playing a role in CML cell immune escape (31–33). The increased numbers of PMN-MDSC, evaluated at diagnosis of CML, return to normal levels after treatment with Imatinib. In acute leukemia, the role of MDSC is not fully defined. Patients with acute myeloid leukemia (AML) display increased numbers of MDSC in PB and BM as compared to patients with acute lymphoblastic leukemia (ALL) and a significant correlation exists with conventional prognostic factors at diagnosis (34). Moreover, a significant decrease of MDSC was observed only in those patients in complete remission after treatment. In pediatric patients, the frequency and the strength of the immunosuppressive function correlated with classical prognostic markers such as MRD and CD20^+^ blast cell counts and with response to therapy. In addition, patients in remission have been reported to lose MDSC suppressive function further corroborating the effect of these cells in favoring immune evasion mechanisms (35, 36). In diffuse large B-cell lymphoma (37), indolent lymphoma (38), chronic lymphocytic leukemia (39), and Hodgkin lymphoma (40), the frequency of circulating MDSC has been correlated with poor prognosis. Recent studies, in the S100A9 knockout transgenic mice, revealed that MDSC are also involved in the pathogenesis and progression of Multiple myeloma (MM) (41). MDSC isolated from the PB of patients with MM display an inhibitory effect on T-cells which could be abrogated by drugs inhibiting arginase-1 and iNOS activity (42). Available data would suggest that MDSC represent a sizable subset present in MM patients that may play a role in the pathophysiology of the disease by favoring survival and proliferation of malignant plasma cells as a consequence of their suppressive activity on anti-tumor immune response. Finally, recent evidence suggests that MDSC have also a predominant role in the pathophysiology of Immune Thrombocytopenia and Chronic Idiopathic Neutropenia (43–45).

### Immunosuppressive Mechanisms Exerted by MDSC

Several studies described different mechanisms adopted by MDSC to exert their immunomodulatory function either by mechanisms that require cell-to-cell contact or by the release of soluble factors. They can directly inhibit the innate or adaptive immune system or indirectly contribute to tumor progression through regulation of angiogenesis or cell motility. In particular, MDSC-derived Nitric oxide (NO) can suppress proliferation of T-cells by inhibiting the Jak/STAT5 pathway and inducing T-cell apoptosis (46). MDSC-derived NO can also impair T-cell migration by reducing E-selectin expression on endothelial cells (47). In addition, oxygen reactive species (ROS) produced by MDSC can induce apoptosis in T-cells by decreasing expression of the T-cell receptor (TCR) ζ-chain (48, 49). Immunosuppression of T-cell response by MDSC may be accomplished through cleavage of L-selectin on CD4^+^ and CD8^+^ T-cells by ADAM metallopeptidase domain 17 (ADAM17) and disintegrin thus impairing T-cell trafficking to tumor sites (50). Moreover, via the ARG-1 or IDO enzymes, MDSC can deprive TME of the amminoacids required by T-cells for proliferation (51–53). Secretion of IL-10 and TGF-β by MDSC represents another mechanism to induce immunosuppression trough Treg induction (54). In lung cancer, IL-10 secreted by M-MDSC has been reported to be in part responsible for Treg induction *in vitro* (55).

Notably, TGF-β and IL-10 can also mediate immunosuppression indirectly, by inducing CD39 and CD73 expression on MDSC that are receptors involved in ATP/ADP hydrolysis and AMP cleavage, respectively, therefore MDSC affect T and NK cell response also by interfering with the adenosine metabolism (56, 57).

Huang B and colleagues showed that IFN-γ secreted by T-cells leads MDSC to release IL-10 and TGF-β that in turn induce Treg (54). In addition to IL-10 and TGF-β, it has been demonstrated that cell-to-cell contact and the CD40 expression on MDSC surface are also required for Treg expansion (58). Contact-dependent mechanism has been demonstrated also in hepatocellular carcinoma where MDSC induce Treg expansion (59). Different factors present in TME (transmembrane TNF-α, TGF-β, lipopolysaccharide, Semaphorin 4D, NKG2D ligands and extracellular vesicles) and hypoxia, can upregulate the secretion of IL-10 by MDSC (60–63). In addition, other factor such as HIF-1α increases the immunosuppressive activity of MDSC by inducing Programmed Cell Death 1 (PD-1) expression and by upregulating the V-domain of Ig suppressor of T-cell activation (VISTA) (64, 65).

Notably, angiogenesis represents another immunosuppressive mechanisms used by MDSC and it is mediated by VEGF upregulation. It has been demonstrated that MDSC, previously activated with VEGF, have a more potent inhibitory activity (66). MDSC can also secrete proangiogenic factors as Metalloproteases (MMP2, MMP8, MMP9, MMP13, and MMP14) that can disrupt the extracellular matrix thus facilitating the extravasation (67).

Another mechanism able to induce immunosuppression is represented by the release of protumorigenic mediators such as S100A8/A9 by MDSC and tumor cells. These factors are capable to induce M2-macrophage polarization and MDSC chemotaxis in TME that results in immunosuppression of effector cells (68, 69).

### NK Cells in Tumors

Natural killer (NK) cells belong to the innate lymphoid cell (ILC) family. ILCs have recently been classified into five different subsets: NK cells that represent killer ILC, and ILC1, ILC2 ILC3, and Lymphoid tissue-inducer cells (LTi) that belong to helper-ILC. Unlike NK cells, the other ILC subpopulations were discovered only recently because they are relatively infrequent and are prevalently located in mucosal tissues and secondary lymphoid organs (70).

NK cells are present primarily in the PB, spleen and bone marrow, but they can infiltrate tissues and are also found in the liver, lungs, gut, lymph nodes and uterus (71–73). Two major subsets of PB-NK cells were identified on the basis of the surface density of CD56 antigen (CD56^bright^ and CD56^dim^). CD56^dim^ NK cells are predominant in PB, display a potent cytolytic activity and release cytokines shortly after receptor-mediated signaling. CD56^bright^ predominate in tissues and secondary lymphoid organs, are poorly cytolytic, while they produce cytokines (74, 75).

The anti-tumor activity of NK cells is primarily related to their cytolytic potential and to the secretion of soluble factors or cytokines that may act on tumors either directly or indirectly upon recruitment of other cell types. NK cell cytotoxicity is induced by surface receptors capable of recognizing ligands that are primarily expressed by tumor cells, but not by most normal resting cells (76). These receptors may induce NK cell activation resulting in tumor cell lysis and secretion of cytokines. The major activating NK receptors include Natural cytotoxic receptor (NCR) (i.e., NKp46, NKp44, and NKp30), DNAM-1 and NKG2D. In addition, NK cells, in most instances, do not kill normal cells thanks to a fail-safe mechanism involving inhibitory receptors specific for HLA-class I molecules. These include killer Ig-like receptors (KIRs) that recognize allotypic determinants of HLA-cl I molecules shared by different groups of alleles and CD94/NKG2A that recognizes HLA-E (77).

During cancer progression, the transformed cells display a decrease or even a loss of the surface expression of MHC-I (78) while strongly upregulate or acquire the expression of ligands for activating NK receptors: two events necessary for NK activation and induction of anti-tumor immune cell responses (79). However, the frequent downregulation of activating receptor expression in NK cells may result in decreased activity leading to increases in tumor expansion and metastases. Indeed, it is well-known that tumor cells may create an immunosuppressive environment through the modulation of inhibitory checkpoints expression on NK cells in order to evade their cytolytic activity and to induce tumor immune escape (79–82).

Some tumors are poorly permeable to NK cells, as the TME may affect their ability to infiltrate the tumor mass. In particular, colorectal carcinoma and melanoma lesions display poor NK cell infiltration (83, 84). On the other hand, NK cell infiltration has been described in other types of tumor and a high number of NK cells in neoplastic tissues has been associated with better survival. For example in breast cancers, tumor-infiltrating (TI) NK cells are used as biomarkers to predict the response to anti-HER2 mAbs therapy (85–87). In the Head and Neck cancers the presence of TI-NK cells correlated with a longer survival (88). Similarly, NK cell infiltration of renal tumors is associated with a good prognosis (85). On the contrary, NK cell infiltration has no impact on clinical outcome in non-small-cell lung cancer (NSCLC) (89, 90).

### NK-MDSC Interactions

NK cells may interact with tumor cells and other cells present in TME through three fundamental pathways, i.e., cell-to cell contact, secretion of soluble molecules in the extracellular milieu, and release of extracellular vesicles (EVs) (91). In cancer patients and tumor mice models, an inverse correlation between the presence of MDSC and NK cells exists (92).

In mice, the mechanism of NK cell inhibition exerted by MDSC is mainly related to cell-to-cell contact and it requires TGF-β (93). Membrane-bound TGF-β on MDSC has been shown to induce NK cell anergy thus impairing their cytotoxic capability and reducing NKG2D expression and IFN-γ production (94). Another study reported IL-33 as a novel player in MDSC-NK interaction. Following stress or damage, IL-33 is secreted by endothelial and epithelial cells and recruits both pro-tumorigenic or anti-tumorigenic immune cells (95, 96).

Furthermore, it has been reported that, in the presence of IL-1β, a novel subset of Ly6C^neg^ MDSC with higher inhibitory properties to NK cells expands in mice (97).

In humans, the inhibition of IFN-γ production by NK cells is related to a NKp30-dependent mechanism (98). MDSC can impair NK cell activity also by interfering with NK FcR-mediated cytotoxicity, as shown in cancer patients NK cells displaying reduced antibody-dependent cytotoxicity and production of cytokines (99).

The IFN-γ and other molecules present in the inflammatory microenvironment are able to promote the expansion of MDSC that, in turn, release high amount of IL-10. IL-10 is considered an anti-inflammatory cytokine capable of inhibiting the release of inflammatory cytokines playing an important role in anti-tumor immunoresponse. In particular, IL-10 may induce a pro-tumorigenic microenvironment affecting both NK cell and CD8^+^cytotoxic T lymphocyte activation and promoting a switch toward type 2 immunoresponse. Targeting either MDSC or IL-10 may favor type1 response and improve the anti-tumor activity of immune cells (100, 101).

Checkpoint blockade immunotherapy targeting the PD-1/PD-L1 inhibitory axis produced remarkable results in the treatment of several types of cancer (102–106). PD-1 is mostly expressed by T-cells, but NK cells with an activated and more responsive phenotype can also express PD-1 (107–110). In TME, tumor cells and their soluble mediators can increase PD-L1 expression on tumor-infiltrating MDSC (111, 112). Thus, PD-L1 expressed by MDSC can suppress NK cell activity while PD-L1 blockade may restore NK and T-cell responses. In different tumor types, increased PD-L1^+^MDSC has been observed and, in some instances, a correlation between the percentage of PD-L1^+^MDSC and disease stages or clinical outcome has been reported (113). In addition, NO produced by MDSC has a potent inhibitory effect on NK cells by impairing the Fc receptor-mediated killing, the secretion of IFN-γ, TNF-α, and Granzyme B, as detected in MDSC-co-cultured NK cells (99). Furthermore, IDO produced by MDSC can impair development and activation of NK cells by decreasing expression of NKG2D, NCR, DNAM1, and IFN-γ secretion (99, 114). IDO production is regulated by STAT3- induced NF-κB activation. It has been demonstrated that blockage of STAT3 and TGF-β can revert the MDSC-mediated inhibition of NK cell function (115, 116).

On the other hand, STAT5 has an opposite effect to that of STAT3. Indeed, STAT5, induced by Jak3, is responsible for perforin, granzyme and IFN-γ production in IL-2 –activated NK cells (117). NK cells co-cultured with MDSC isolated from spleen of tumor-bearing mice, showed both Jak3-inhibition and reduced STAT5 activation (98).

Another mechanism occurring in NK-MDSC interaction involves the TIGIT-CD155 axis. Thus, analysis of patients with CMV^+^ myelodysplastic syndrome revealed the presence of adaptive NK cells with lower TIGIT expression (partially) resistant to MDSC-mediated immunosuppression (118). Recently, in NK cells the IL-1R8 has been suggested as a novel immune check-point that can potentially interact with MDSC cells (119).

### MDSC and NK Cross Talk in HSCT

MDSC were originally described as cells able to inhibit T cell activation, proliferation, and function. Other studies provided evidences that MDSC could also interact and interfere with the function of other cells, including NK cells, B cells, NKT cells, and DCs. All these observations are in line with the suppressive effect of MDSC in the context of hematopoietic stem cell transplantation (HSCT). Notably, HSCT from HLA-matched donor, either related or unrelated, is extensively used to cure patients with Acute Leukemia. The HSCT from HLA-haploidentical relatives (haplo-HSCT) gave the opportunity of a prompt transplantation in patients with no HLA-matched donor. Graft-vs.-host disease (GvHD) and post-transplant lymphoproliferative disease (PTLD) are two life-threatening effects of un-manipulated HSCT, due to the presence of T cells and B cells in the graft. In haplo-HSCT, graft of “mega-doses” of highly purified CD34^+^ HSC has been applied for many years. However, the lack in the graft of different mature lymphoid subsets and of (CD34^−^) committed hematopoietic progenitors results in a prolonged lymphopenia and delayed immune reconstitution that causes an increased risk of non-relapse-related mortality (NRM), due primarily to opportunistic infections. Thus, selective depletion of αβ T lymphocytes, and of B cells was used more as a novel method of graft manipulation. This approach allows the infusion in the recipient not only of hematopoietic progenitors but also of high numbers of donor mature NK cells, γδ T-cells and myeloid cells. In particular, NK and γδ T-cells transferred with the graft may contribute to prevent leukemia relapses and severe viral infections and/or reactivation before the establishment of adaptive immune responses thanks to their activity against leukemia blasts remaining in the patient after the conditioning regimen (120). Notably, it has been shown that in αβ T- and B-cell depleted HSCT setting, the contribution of the NK cell alloreactivity to the 5 years' survival probability was partially obscured, possibly by the effect of γδ T-cells (121–123).

**Figure 1:**
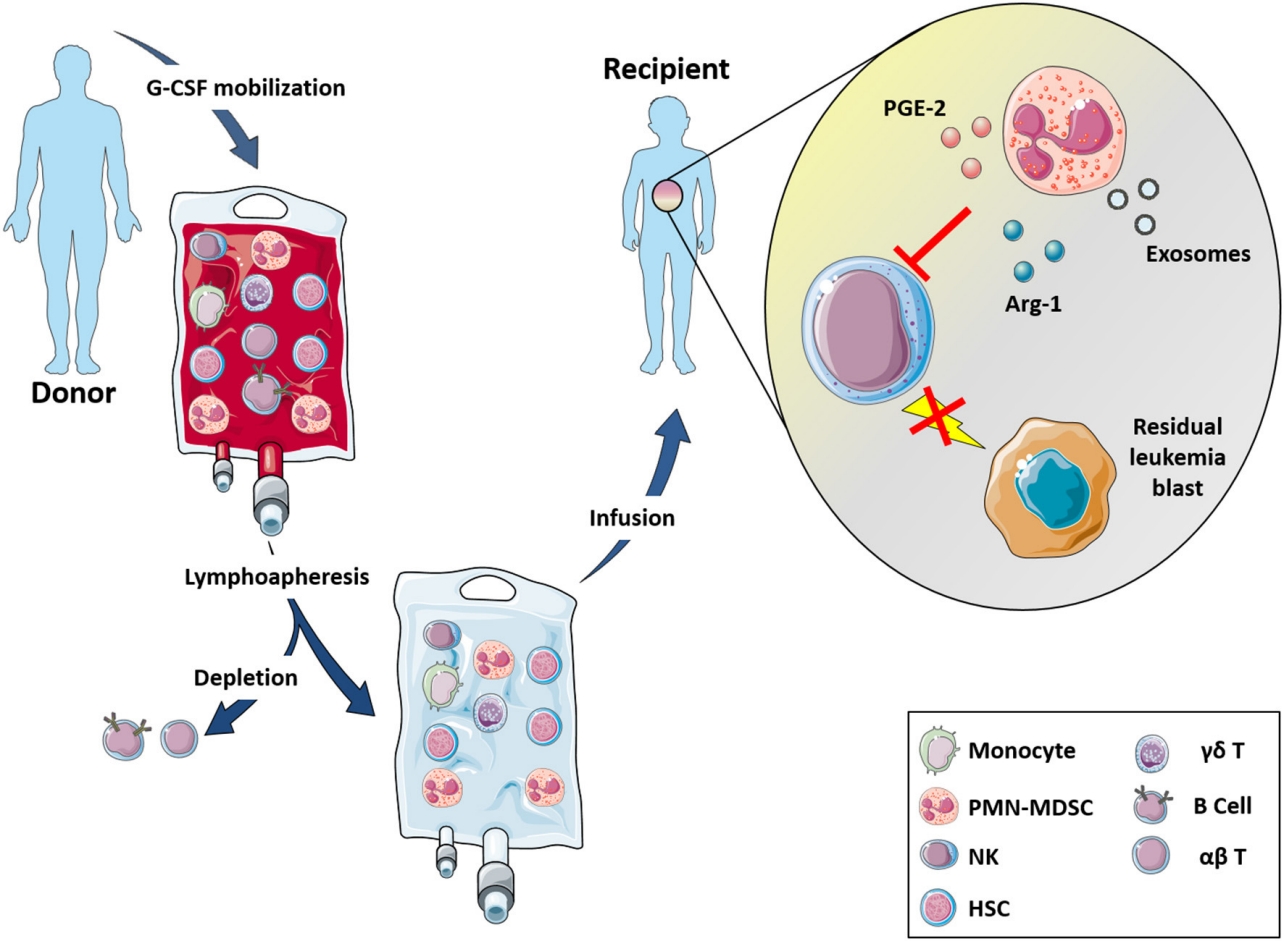
αβ T- and B-depleted hematopoietic stem cell transplantation protocol. Donors undergo lymphoapheresis after 5 days G-CSF stimulation for increasing the number of HSC cells and to induce PMN-MDSC mobilization. Thus, αβ T and B cells are depleted and infusion (enriched in NK cells, γδ T-cells and myeloid cells) is administered to recipient patients. In this transplant setting, the enrichment of PMN-MDSC helps to reduce NK cell cytotoxic effects against the graft by the release of IDO2, PGE2, and exosomes thus suggesting a possible involvement of these myeloid cells into the GvL activity mediated by NK cells.

Regarding the strategy routinely applied to increase the number of circulating HSC to be infused, donors receive G-CSF for 5 days (Figure 1). G-CSF induces a proteolytic microenvironment and inhibits CXCL12 production, thus favoring HSC egress from BM. An adequate number of HSC can be achieved also in “poor mobilizer” donors, who, in addition to G-CSF, receive Plerixafor (PL), a CXCR4 antagonist, which inhibits HSC retention in the BM, favoring their collection in the peripheral blood (PB). The G-CSF mobilization regimen induces an accumulation in PB of PMN-MDSC (124).

We could demonstrate that PMN-MDSC derived from G-CSF mobilized donors did not interfere with the differentiation of donor HSC (124). On the contrary, they could affect the cytotoxic potential of donor-mature NK cells, which are infused into the patients during transplantation, compromising their GvL activity.

PMN-MDSC through the release of IDO metabolites and PGE2 down modulate the expression of intracellular polypeptides involved in the signal transduction and of the major activating NK receptors. In particular, signaling via activating receptor is mediated intracellularly by immunoreceptor tyrosine-based activation motifs (ITAM) and by downstream protein kinases. KARAP/DAP12 and CD3ζ are ITAM-bearing adaptor proteins known to associate with different activating NK receptors. These intracellular molecules involved in signal transduction were down-modulated in NK cells upon interaction with PMN-MDSC.

PMN-MDSC were also shown to affect NK cell degranulation, cytokine release and cytotoxicity. Indeed, in the presence of IDO- and PGE2-inhibitors the NK-cell activity can be recovered suggesting the involvement of IDO catabolites and PGE2 in the inhibition of the NK-mediated killing of leukemia blasts (124). It is known that MDSC may exploit additional immunomodulatory mechanisms including, for example, the release of exosomes. In this context, PMN-MDSC were able to release exosomes that are, in turn, internalized into NK cells and cause an impairment of their cytolytic activity (125) (Figure 1). Altogether, these data indicate that PMN-MDSC exert a potent inhibitory effect on anti-tumor NK cell function suggesting their possible involvement in the impairment of GvL activity mediated by NK cells.

Based on the *in vitro* data and on the role of these cells in hematologic malignancies, it is conceivable that MDSC may indeed represent a key immunosuppressive cell type induced in allogeneic HSCT. Further investigation regarding molecular and functional characteristics of MDSC may help to discover new strategies/drugs, to either dampen or enhance MDSC immunosuppressive activity, depending on the therapeutic need in different clinical contexts.

## Concluding Remarks

A deeper comprehension of the mechanisms and relative molecular pathways adopted by MDSC present in TME to impair the anti-tumor function of immune effector cells may allow to identify novel therapeutic strategies capable to disrupt these potent inhibitory mechanisms. Thus, in HSCT the large proportion of PMN-MDSC can counteract the GvL activity mediated by donor-mature NK cells infused in the recipient, particularly in the early post-transplant period. Previous reports revealed that a reduction of the immunosuppressive activity of MDSC could be achieved by inducing their differentiation. It has been reported that the combined administration of ATRA (all-trans-retinoic acid) (126), paclitaxel (ultra-low non-cytotoxic doses) (127), vitamin D (128), and IL-2 (129) is able to induce MDSC differentiation by blocking their immunosuppressive activity and resulting in the recovery of immune response. *In vitro* data showed that chemotherapeutic agents (i.e., gemcitabine or 5-fluorouracil) could be used to selectively deplete MDSC with no toxic effects on other leukocyte populations (130, 131).

A better outstanding of interactions occurring between NK cells and PMN-MDSC, in particular in TME, may offer an interesting clue to further improve the efficacy of immunotherapy. In particular, in αβ T- and B cell-depleted haplo-HSCT setting, removing also PMN-MDSC, could preserve the NK-cell function with a further positive effect on the GvL activity and viral protection, obtaining a better patient's clinical outcome.

## Author Contributions

All authors listed have made a substantial, direct and intellectual contribution to the work, and approved it for publication.

## Conflict of Interest

The authors declare that the research was conducted in the absence of any commercial or financial relationships that could be construed as a potential conflict of interest.
